# γ-Carboxymuconolactone decarboxylase: a novel cell cycle-related basal body protein in the early branching eukaryote *Trichomonas vaginalis*

**DOI:** 10.1186/s13071-017-2381-4

**Published:** 2017-09-26

**Authors:** Wei-Hung Cheng, Kuo-Yang Huang, Po-Jung Huang, Chi-Ching Lee, Yuan-Ming Yeh, Fu-Man Ku, Rose Lin, Mei-Ling Cheng, Cheng-Hsun Chiu, Petrus Tang

**Affiliations:** 1 Graduate Institute of Biomedical Sciences, College of Medicine, Chang Gung University, Kweishan, Taoyuan, Taiwan; 2 Molecular Regulation and Bioinformatics Laboratory, Department of Parasitology, College of Medicine, Chang Gung University, Kweishan, Taoyuan, Taiwan; 3 Graduate Institute of Pathology and Parasitology, National Defense Medical Center, Taipei, Taiwan; 4 Bioinformatics Core Laboratory, Molecular Medicine Research Center, Chang Gung University, Kweishan, Taoyuan, Taiwan; 5 Department of Biomedical Sciences, College of Medicine, Chang Gung University, Kweishan, Taoyuan, Taiwan; 6 Department and Graduate Institute of Computer Science and Information Engineering, College of Engineering, Chang Gung University, Kweishan, Taoyuan, Taiwan; 7 Molecular Infectious Disease Research Center, Chang Gung Memorial Hospital, Kweishan, Taoyuan, Taiwan

**Keywords:** γ-carboxymuconolactone decarboxylase, *Trichomonas vaginalis*, Iron deficiency, Basal body, Cell cycle, Lateral gene transfer

## Abstract

**Background:**

γ-Carboxymuconolactone decarboxylase (CMD) participates in the β-ketoadipate pathway, which catalyzes aromatic compounds to produce acetyl- or succinyl-CoA, in prokaryotes and yeast. Our previous study demonstrated that expression of a CMD homologue that contains two signatures (dualCMD) is negatively regulated by iron in *Trichomonas vaginalis*. However, we were not able to identify the components of the β-ketoadipate pathway in the parasite’s genome. These observations prompted us to investigate the biological functions of this novel CMD homologue in *T. vaginalis*.

**Methods:**

The specific anti-TvCMD1 antibody was generated, and the expression of TvCMD1 in *T. vaginalis* cultured under iron-rich and iron-deficient were evaluated. Phylogenetic, metabolomic and substrate induction (protocatechuate and benzoate) analysis were conducted to clarify the function of dualCMD in trichomonad cells. Subcellular localization of TvCMD1 was observed by confocal microscopy. The cell cycle-related role of TvCMD1 was assessed by treating cells with G2/M inhibitor nocodazole.

**Results:**

We confirmed that *T. vaginalis* is not able to catabolize the aromatic compounds benzoate and protocatechuate, which are known substrates of the β-ketoadipate pathway. Using immunofluorescence microscopy, we found that TvCMD1 is spatially associated with the basal body, a part of the cytoskeletal organizing center in *T. vaginalis*. TvCMD1 accumulated upon treatment with the G2/M inhibitor nocodazole. Additionally, TvCMD1 was expressed and transported to/from the basal body during cytokinesis, suggesting that TvCMD1 plays a role in cell division.

**Conclusion:**

We demonstrated that TvCMD1 is unlikely to participate in the β-ketoadipate pathway and demonstrated that it is a novel basal body-localizing (associated) protein. This model sheds light on the importance of genes that are acquired laterally in the coevolution of ancient protists, which surprisingly functions in cell cycle regulation of *T. vaginalis*.

**Electronic supplementary material:**

The online version of this article (10.1186/s13071-017-2381-4) contains supplementary material, which is available to authorized users.

## Background

Aromatic compounds (such as benzoate) are frequently used as preservatives, which are found in natural pollutants and vascular plants [1, 2]. Some bacteria possess the β-ketoadipate degradation pathway to digest these hazardous compounds and provide alternative carbon sources for energy production via the tricarboxylic acid cycle. γ-carboxymuconolactone decarboxylase (CMD) is a key enzyme of this pathway that converts γ-carboxymuconolactone to β-ketoadipate-enol-lactone [3]. A group of CMDs composed of two CMD signatures (referred to dualCMD) has been identified in bacteria, archaea and eukaryotes. However, very little information is available about the biological functions of these dualCMDs.

Genes derived from lateral gene transfer (LGT) play critical roles in metabolic and pathogenic regulation in *Trichomonas vaginalis*, the causal agent of the most prevalent non-virally sexual transmitted disease worldwide [4–7]. For instance, glycan breakdown pathway with enzymes of bacterial origin is crucial for colonization [8]. Approximately ~0.25% of protein-coding genes in *T. vaginalis* are considered as LGT origin, but few have been investigated [9]. According to next generation sequencing-based gene expression analysis, one highly-expressed trichomonad dualCMD (TvCMD1) appears in iron-deficient cultivation [10]. A survey of the genome of *T. vaginalis* indicated that there were no putative genes from the β-ketoadipate pathway [9]. Thus, either the protist is likely to employ different enzymes from the known components for aromatic compound catabolism or TvCMD1 has evolved a new function distinct from the original one.

Iron is involved in many processes inside cells that are essential for establishing *T. vaginalis* infection. Previous studies have suggested that the cytotoxicity, adherence and immune evasion capacities of the protist decreased in cells grown in iron-deficient environments [11–13]. In addition, insufficient iron supplementation slows the proliferation of *T. vaginalis*, eventually resulting in cell death [10, 14]. Indeed, iron limitation leads to cell cycle arrest at either the G1 or G2/M phases in mammalian cells, and thus, the fluctuation of the iron concentration throughout the menstrual cycle is challenging for the proliferation of *T. vaginalis* in the vaginal region [15, 16]. The basal body, a part of the cytoskeletal organizing center, is involved in not only flagellum assembly but also spindle pole and axostyle-pelta complex formation, and that is thought to be the critical proteinaceous structure for the primitive and closed mitotic cell cycle in this protist [17–19]. However, the molecular mechanism of cell cycle regulation in *T. vaginalis*, particularly during iron deficiency, is still unknown.

In the present work, we characterized TvCMD1 using a multi-omics approach. A database survey suggested that dualCMD was broadly distributed in organisms, as in the cases of *Giardia intestinalis* and *T. vaginalis*. According to phylogenetic, protein induction and metabolomics assays, TvCMD1 was unlikely to be involved in the β-ketoadipate pathway. TvCMD1 was found to be associated with the basal body in *T. vaginalis*. Moreover, TvCMD1 was expressed and transferred to/from the basal body in the G2/M phase, reflecting a role for TvCMD1 in cytokinesis. This study provides an example of a bacterial origin gene that performs an unrelated function in the early branching protist *T. vaginalis*.

## Methods

### Cell culture and treatments

*Trichomonas vaginalis* ATCC isolate 30,236 was cultured at 37 °C in yeast extract iron-serum (YI-S) medium containing 80 μM ferrous ammonium citrate (Sigma-Aldrich, Merck, Darmstadt, Germany) (iron-rich condition) [20]. Iron-deficient cells were grown in YI-S medium without iron supplementation, and 180 μM of the iron chelator dipyridyl (DIP, Sigma-Aldrich, Merck) was added at a cell density of 10^6^ cells/ml [10]. Cells for assays were harvested from the mid-logarithmic phase of iron-rich cells, and the iron-deficient cells were cultured with DIP for at least 6 h. The trypan blue exclusion assay was used to monitor the growth of cells. Mid-logarithmic phase trichomonad cells were collected and treated with nocodazole (10 uM) and incubated for 0, 3, 6 and 9 h at 37 °C after cell cycle synchronization by cold incubation [21].

### Sequence and phylogenetic analysis

We generated a dataset of 1202 dualCMD sequences based on the sequence homology of TvCMD1 (TVAG_256720) and the presence of dualCMD domain signatures (UniProt database) for phylogenetic analysis (Additional file 1: Table S1) [22]. Redundant sequences were trimmed (sequences ≥ 80% identity were deleted and kept one randomly) and 540 protein sequences were retained by the CD-HIT tool [23]. The sequences were aligned using the MAFFT algorithm with default parameters [24]. The Bayesian analysis was performed in Phylobayes 4.1 with two Monte Carlo Markov Chains (MCMC) using the LG model with catfix C20 and poisson [25–28]. The chains were stopped until convergence (maxdif <0.3) for each chain. The MCMC was estimated by sub-sampling every 10 trees after discarding the first 1000 ‘burn-in’ trees.

### Quantitative real-time polymerase chain reaction

mRNA was extracted and reverse-transcribed to cDNA as described previously [10]. Quantitative real-time PCR was performed to verify the expression of three TvCMDs in cells cultured under iron-rich and iron-deficient conditions [29]. The reaction mixture was composed of 1 μg of cDNA, double-fold master mix (Amplicon, Brighton, United Kingdom), 0.5 μM primer, and filled to 20 μl with deionized water. The primer sets used in this experiment are listed in the Additional file 2: Table S2.

### Cloning, expression, and antibody generation of TvCMD1

The coding sequence of TvCMD1 was obtained from the genome database TrichDB. The full-length TvCMD1 DNA sequence was amplified from the genomic DNA of ATCC30236 by PCR using a specific primer set (forward: 5′-ATG AGT TAC AAA GCG ACG GAA CA-3′; reverse: 5′-TTA TTC ATC AGG GAT GGC GTC ATT-3′). The PCR product was cloned into the pTrcHis TOPO TA cloning vector (Invitrogen, Thermo Fisher Scientific, Waltham, MA, USA) and transformed into competent cells (Top 10 cells, Invitrogen, Thermo Fisher Scientific). The clone with the correct orientation and nucleotide sequence was induced by isopropyl β-D-1-thiogalactopyranoside (IPTG) for protein expression. After a 4 h induction, bacteria were lysed, and soluble protein was extracted by sonication. Recombinant TvCMD1 in the total protein was purified using a nickel column, and the TvCMD1 concentrated fractions were collected and used as the immunogen for antibody generation (Abomics, New Taipei City, Taiwan).

### Whole-cell protein extraction

Cells (2 × 10^7^) were obtained from the mid-logarithmic phase of iron-rich and 6-h iron-deficient cultivations. After the wash steps, cell pellets were suspended in NP-40 lysis buffer (5 mM magnesium chloride, 100 mM sodium chloride, 20 mM Tris-HCl (pH 7.4), 0.5% NP-40, 10 μg/ ml Aprotinin, 1 mM 4-(2-Aminoethyl) benzenesulfonyl fluoride hydrochloride, 5 mM levamisole, 0.7 mM sodium orthovanadate), mixed by vortexing, incubated on ice for 20 min and centrifuged for 20 min at 4 °C. The supernatant (water-soluble fraction) was harvested from each sample, and the concentration was determined by the Bradford protein assay.

### Immunoblotting

The calculated amount of soluble protein was mixed with sample buffer (0.5% bromophenol blue, 0.1 M dithiothreitol, 10% glycerol, 2% sodium dodecyl sulfate, 0.5 M Tris-hydrochloride) and boiled for 5 min at 95 °C. The prepared samples were separated by 12% sodium dodecyl sulfate polyacrylamide gel electrophoresis (SDS-PAGE) and transferred to a nitrocellulose membrane. The transferred membrane was washed with TTBS (Tris-buffered saline and Tween 20) and blocked with 5% milk for 1 h at room temperature. Hybridization of the membrane with primary antibodies (anti-TvCMD1 and anti-GAPDH, 1:2000) occurred with incubation at 4 °C overnight. The membrane was washed three times with TTBS and incubated with secondary antibodies (horseradish peroxidase-conjugated antibodies) for 40 min at room temperature. After the washing steps, the membrane was incubated with the HRP substrate, and the signals were visualized using a UVP BioSpectrum 600 Imaging system (UVP, CA, USA).

### Aromatic compound induction assay

Iron-rich cultured mid-logarithmic phase trichomonads were collected for aromatic compound treatment. Approximately 2 × 10^6^ cells were seeded in 2.5 ml of the medium that contained 5 mM protocatechuate or benzoate and incubated at 37 °C for 3 h. Sterile water instead of aromatic compounds was used as the control. The protein expression level of the TvCMD1 whole cell lysate was determined by western blotting using an anti-TvCMD1 antibody.

### Measurement of benzoate and protocatechuate using ultra-performance liquid chromatography-tandem mass-spectrometry (UPLC-MS/ MS)

Intracellular protocatechuate and benzoate were detected as previously described [30]. Cells (2 × 10^6^) cultured under iron-rich and iron-deficient conditions were harvested. The cell pellets were washed with cold PBS, resuspended in cold 80% methanol and kept at -80 °C overnight. Cells were collected via centrifugation at 14,000× *g* for 30 min at 4 °C. The supernatant was transferred to new micro-centrifugation tubes and dried under nitrogen gas. The residue was dissolved in 90 μl of 10 mM ammonium formate (pH 8.0) for downstream UPLC-MS/ MS analysis. The Acquity UPLC system (Waters Corporation, Milford, Massachusetts, USA) was composed of a binary solvent manager, a vacuum degasser, a column heater and sample manager. The column temperature was maintained at 45 °C. The samples were inserted into an Acquity UPLC BEH C18 column, 100 × 2.1 mm, 1.7 μm particle diameter (Waters Corporation). Protocatechuate and benzoate were separated by a linear gradient of solution A (0.05% formic acid) and B (acetonitrile containing 0.05% formic acid). The flow rate was 0.5 ml/ min, and the gradient was as follows: 0–5 min, 1% B; 5 min, 30% B; 5.01–10 min, 1% B. Two microliters of each sample was analyzed, and the total running time was 8 min. A Xevo TQMS (Waters Corporation) was used in the negative electrospray ionization mode. Nitrogen was used as the desolation gas (800 l/h) and cone gas (150 l/h). The cone voltage was 2 V, and the collision energy was 12 eV. The capillary voltage was set at 2 kV, and the source temperature was 150 °C. Protocatechuate and benzoate were detected in multiple reaction monitoring (MRM) mode with a dwell time of 0.045 s. Based on the standard compounds, the parent and product ions (m/z) of protocatechuate were 153.09 and 109.07, and for benzoate were 121.03 and 77.08.

### Immunofluorescence assay

Iron-rich cells and cells treated with DIP for 6 h were collected and spread on the slides. After air drying for 5 min, a fixing solution (4% formaldehyde) was added, and the slides were incubated for 20 min at room temperature. The slides were washed with PBS and permeabilized with 0.1% Triton X-100/PBS for 10 min at room temperature. After a 1 h blocking reaction, the blocking buffer (3% bovine serum albumin (BSA)) was discarded and the slides were incubated with anti-TvCMD1 and -centrin primary antibodies (clone 20H5, Millipore, Merck, Darmstadt, Germany) (1:500 in 3% BSA- 0.1% Triton X-100) for 1 h at room temperature. The slides were washed with PBS and incubated with fluorescent secondary antibodies (anti-rabbit and anti-mouse IgG antibodies for TvCMD1 and centrin, respectively; 1:500 in 3% BSA) for 1 h at room temperature. Cells were washed with PBS and stained with 4′,6-diamidino-2-phenylindole (DAPI, 0.1 μg/ ml, Sigma-Aldrich, Merck, Darmstadt, Germany) for 15 min at room temperature. After staining, the slides were washed with PBS and air-dried for 5 min. Mounting solution (0.17 M KHCO3, 50% glycine) was added to the slides, and the slides were sealed with coverslips for confocal microscopy examination (Zeiss LSM510).

### Cell cycle analysis

The cell cycle of *T. vaginalis* was analyzed by flow cytometry as follows. Approximately 2 × 10^6^ cells were collected and washed with cold PBS. The pelleted cells were fixed in 70% ethanol and incubated at -20 °C for 1 h. After removal of the fixing solution, the cells were incubated in buffer containing 0.5% Triton X-100 and 0.05% RNase at 37 °C for 1 h. Propidium iodide (PI, 50 μg/ ml) was added to the cells and stained at 4 °C for 20 min. The analysis was performed using a Becton Dickinson FACSCalibur flow cytometer (Becton, Dickinson and Company (BD), New Jersey, USA). A total of 10,000 cells were acquired, and the DNA content of each group was represented as histograms and analyzed by using ModFit LT software (version 3.3.11).

### Statistical analysis

Student’s t-tests were performed on the quantified data derived from biological assays using GraphPad Prism 5 software. Asterisks represent the significance of each assay as determined by the *P*-value (*P < 0.05; **P < 0.01; ***P < 0.001).

## Results

## Sequence analysis of TvCMD1

TvCMD1 is a dualCMD composed of two CMD signatures and twice the length of canonical CMDs. Three dualCMD paralogs were identified in the genome of *T. vaginalis*, sharing 68% and 40% identity of TvCMD1 (TVAG_256720) to TvCMD2 (TVAG_107080) and TvCMD3 (TVAG_474690), respectively (Additional file 3: Figure S1a).

Previous studies demonstrated that non-canonical CMDs serve as disulfide reductase- and thioredoxin-like proteins [31, 32]. To examine the probable function of TvCMD1, we divided the TvCMD1 sequence into two CMD signatures and aligned them with these CMDs. As shown in Additional file 3: Figure S1b, neither the functional cysteine residues of the iron-sulfur cluster binding sites (brown box) nor CXXC domains (blue box) were found in both the first and second CMD signatures of TvCMD1. Hence, it is unlikely that TvCMD1 exerts electron transfer-related activities.

### Iron negatively regulated the expression of TvCMD1 in *T. vaginalis*

**Fig. 1:**
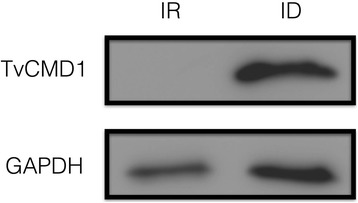
Expression of TvCMD1 in different iron availabilities. Protein levels of TvCMD1 under iron-rich (80 μM FAC) and iron-deficient (180 μM DIP) conditions as detected by western blot. GAPDH was used as the loading control. This result indicated a negative iron regulation of TvCMD1 at protein level

Our previous report revealed that the mRNA level of TvCMD1 (TVAG_256720) was elevated when cells were cultured under iron-deficient conditions [10]. Using qPCR analysis, it was confirmed that TvCMD1 was the most abundant among the three dualCMDs under iron-deficient conditions (Additional file 4: Figure S2). The protein level of TvCMD1 in trichomonad cells was also determined using an anti-TvCMD1 antibody (Additional file 5: Figure S3), showing that a ~26-kDa band was exclusively expressed under iron-deficient conditions, whereas no signal could be detected in the iron-rich control group (Fig. 1). These data indicated that upregulation of the TvCMD1 protein is the same as for mRNA under iron-deficient conditions.

### TvCMD1 may not function in β-ketoadipate pathway upon iron deficiency in *T. vaginalis*

A phylogeny was built to illustrate the evolutionary history and functional relevance among dualCMDs. Proteobacteria, Actinobacteria, Bacteroidetes, and Firmicutes bacteria were the major members in this phylogenetic tree (Additional file 6: Figure S4). Fungal dualCMDs were distributed separately: dualCMDs of *Alternaria alternate*, *Botryosphaeria parva* and *Zymoseptoria brevis* were clustered with Proteobacteria, whereas another dualCMD from *Gonapodya prolifera* was categorized far from the fungal groups (clades with yellow background). Organisms considered to be capable of degrading aromatic compounds (protocatechuate 3,4-dioxygenase α and β subunits (P3,4O)-encoding, the first enzyme of the β-ketoadipate pathway, asterisk [3]) were mainly grouped in the phyla Proteobacteria and Actinobacteria, although some bacteria that belong to Bacteroidetes also encoded P3,4O. In addition, all analyzed Archaea (green) and fungi contained both CMD (circles) and dualCMD, but no P3,4O. Flagellate dualCMDs (*T. vaginalis* and *G. intestinalis*) were closely associated with the phyla Firmicutes and Actinobacteria (clade with gray background), suggesting that they shared an evolutionary origin. Notably, no bacteria in this clade were likely to degrade aromatic compounds since they do not encode P3,4O. Accordingly, we speculated that dualCMDs in this clade might play roles other than aromatic compound degradation.

**Fig. 2:**
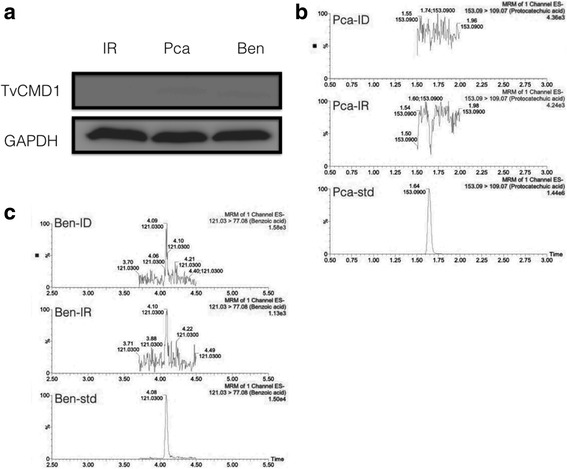
TvCMD1 does not participate in β-ketoadipate degradation pathway. **a** Aromatic compounds induction assay in *T. vaginalis*. Western blot was used to monitor the expression of TvCMD1 after incubating with protocatechuate (Pca) and benzoate (Ben) for 3 h. GAPDH was used as the loading control. **b**, **c** The detection of aromatic compounds in trichomonad cell lysates. The parent and product ions (m/z) ratio was defined according to multiple reaction monitoring (MRM) of each standard. The quantification of aromatic compounds (protocatechuate, benzoate) in iron-rich (middle panel, IR) and iron-deficient (upper panel, ID) cultured *T. vaginalis*. Percentage (Y-axis) represents the relative concentration of each compound. The lower panels are the standards (std, 1 ppm) Pca and Ben. The protein induction assay and metabolites detection showed that TvCMD1 is unlikely to catalyze aromatic compounds protocatechuate and benzoate

The previous study revealed that CMD expression could be induced by substrates of the β-ketoadipate pathway [33]. To determine whether TvCMD1 participates in this pathway, we treated trichomonad cells with protocatechuate and monitored the expression of TvCMD1 in iron-rich culture (absence of TvCMD1 protein). The protein level of TvCMD1 exhibited no significant change in cells treated with protocatechuate (Pca) (Fig. 2a). Similarly, benzoate (Ben) could not induce the expression of TvCMD1.

To further clarify whether the expression of TvCMD1 depends on the presence of protocatechuate or benzoate in *T. vaginalis*, we utilized a metabolomics approach to detect these compounds in the cell lysates of iron-rich and iron-deficient groups. In Fig. 2b, protocatechuate is undetectable in both groups. A comparable consequence was observed for benzoate levels (Fig. 2c). These data demonstrated that TvCMD1 expression is not related to the accumulation of protocatechuate or benzoate. In other words, the function of TvCMD1 may be not related to the catabolism of aromatic compounds in *T. vaginalis*.

### Colocalization of TvCMD1 with the basal body and the associated cytoskeletal structures in *T. vaginalis*

**Fig. 3:**
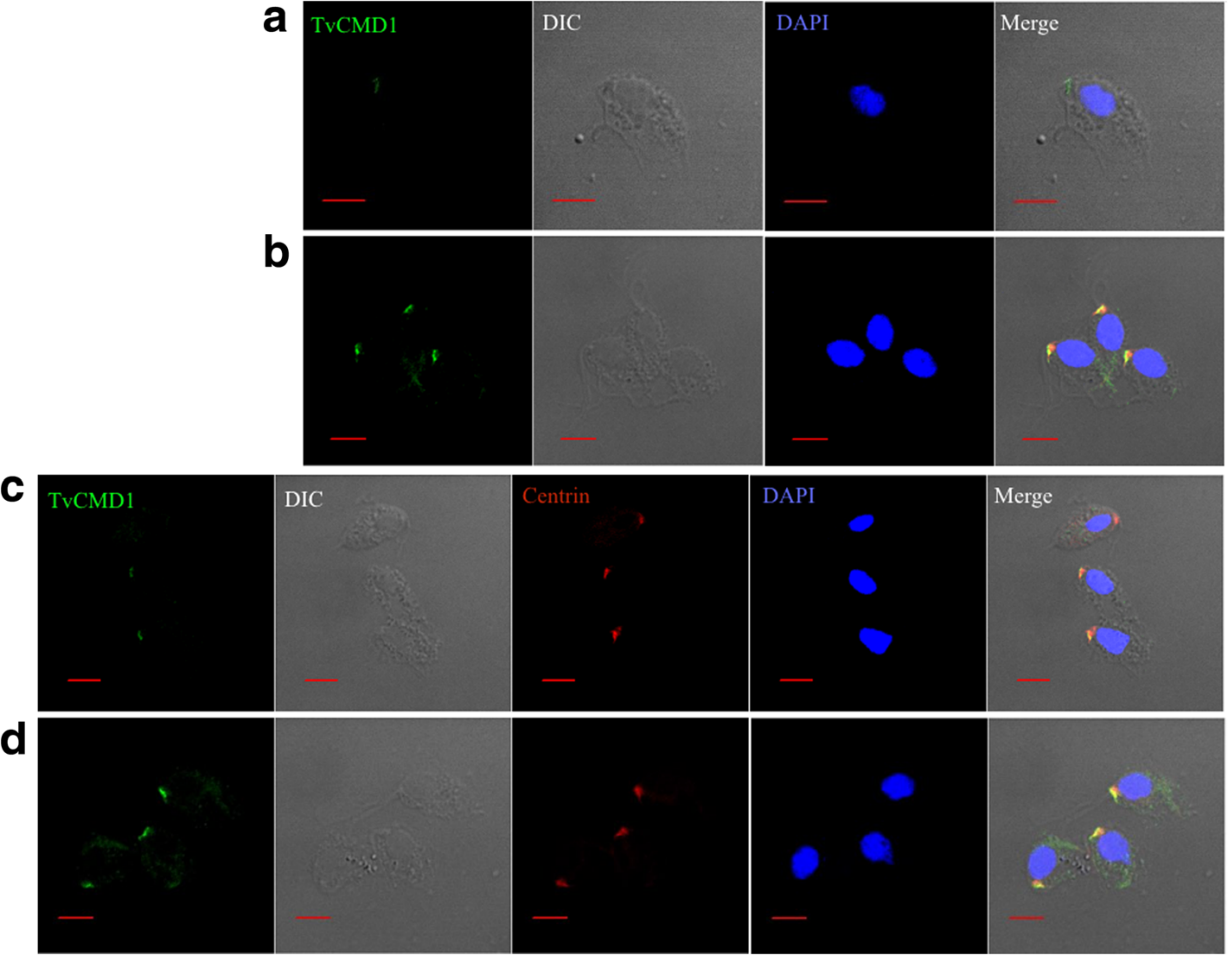
TvCMD1 is associated with the basal body in *T. vaginalis*. Immunofluorescence analysis shows anterior localized TvCMD1 (green fluorescence) in iron-rich (**a**) and iron-deficient (**b**) cultured cells. TvCMD1 co-localized with basal body marker centrin (red fluorescence) in iron-rich (**c**) and iron-deficient (**d**) cells. The nucleus was stained with DAPI (blue fluorescence). The result indicated that TvCMD1 (green color) was colocalized with the basal body (red color) in *T. vaginalis*. *Scale-bars*: 5 μm

To investigate the role of TvCMD1 in *T. vaginalis* upon iron deficiency, we determined the subcellular localization of this protein using an immunofluorescence assay (IFA). The TvCMD1 signal was almost absent in iron-rich cultured trichomonads, with only a weak signal observed in a few examined cells (Fig. 3a). By contrast, an abundant TvCMD1 signal appeared in the same region in cells cultured under iron-deficient conditions (Fig. 3b). The fluorescence intensities of TvCMD1 under iron-rich and iron-deficient conditions are similar to that of the immunoblotting data (Fig. 1), reaffirming the iron-dependent expression of TvCMD1 in *T. vaginalis*.

The anterior-localized TvCMD1 raised the possibility that TvCMD1 may be associated with the basal body, flagella forming center and a part of the cytoskeletal organizer in *T. vaginalis*. To address this hypothesis, we used centrin as a marker to monitor the spatial relationship between the basal body and TvCMD1 [34]. Colocalization of TvCMD1 and centrin was observed under both iron-rich and iron-deficient conditions (Fig. 3c, d), confirming that TvCMD1 localized on the basal body in *T. vaginalis*.

### TvCMD1 is a cell division associated protein in *T. vaginalis*

**Fig. 4:**
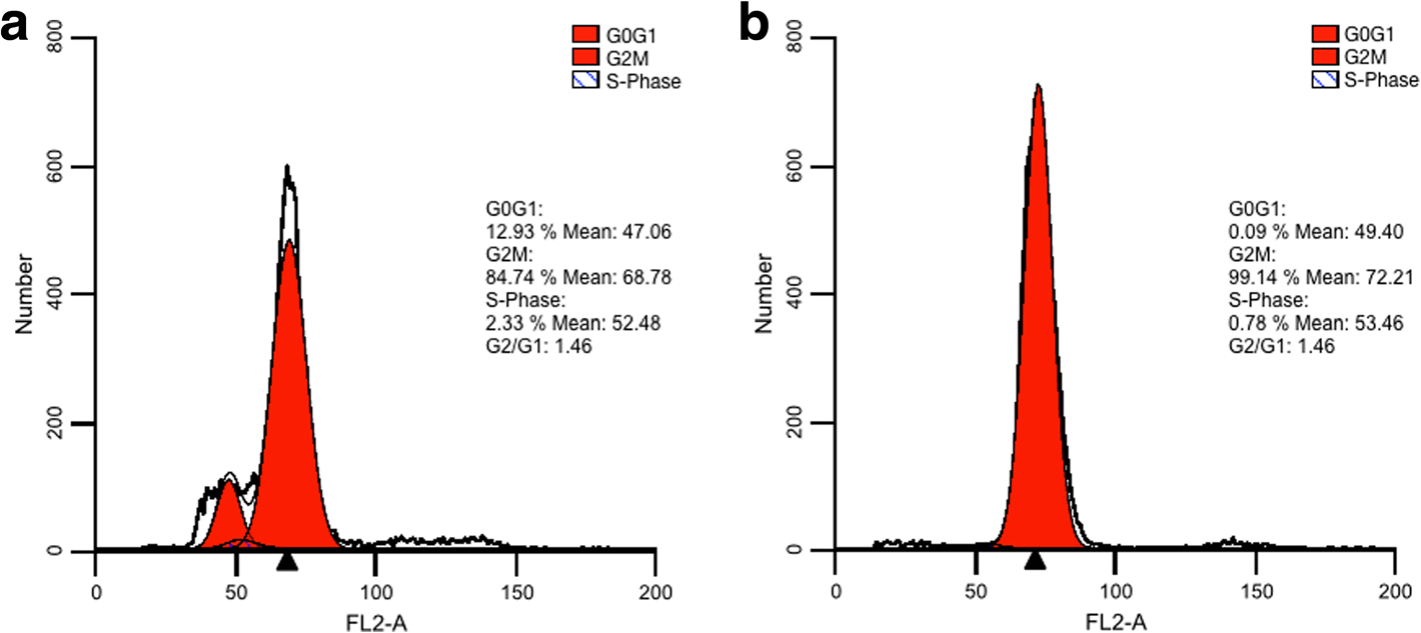
Iron deficiency induces G2/M arrest in *T. vaginalis*. Cell cycle stage of *T. vaginalis* cultured under iron-rich (**a**) and iron-deficient (**b**) conditions were assessed by flow cytometry. DNA was stained with propidium iodine (PI) and shown in histograms. The G2/M (arrowhead) phase was indicated. This result indicated that iron deficiency induces G2/M arrest in *T. vaginalis*

**Fig. 5:**
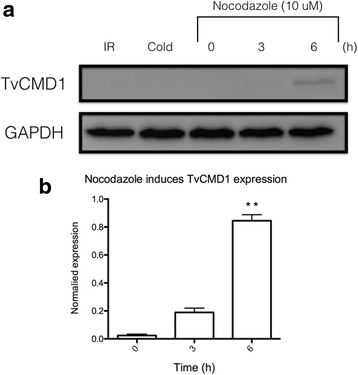
Nocodazole induces expression of TvCMD1 in a time-dependent manner. **a** Western blotting revealed the expression of TvCMD1 after nocodazole (10 μM) treatment for 0, 3 and 6 h. The iron-rich cultured cells were synchronized by cold incubation for 3 h prior to nocodazole addition (cold). GAPDH was used as the loading control. **b** The quantified result of nocodazole-treated groups based on penal (**a**). Asterisks represent the significance of 6 h compared to 0 h as determined through *P*-value (**P < 0.01). The expression of TvCMD1 could be induced by the treatment of nocodazole, the G2/M inhibitor. *Abbreviation*: IR, iron-rich (negative control)

The basal body is the crucial proteinaceous structure that regulates cell cycle progression in *T. vaginalis* [19, 34, 35]. Flow cytometry indicated that iron deficiency reduces the number of G1/S phases cells and increases the number of G2/M cells in *T. vaginalis* (Fig. 4). In addition, the expression of TvCMD1 mRNA was upregulated upon stresses that extended the stationary phase of *T. vaginalis*, such as cold treatment, glucose restriction and oxygen stress [29, 36–39]. We thus hypothesized that the expression of TvCMD1 might be linked to cell cycle progression, especially the G2/M phase. The cells were treated with the G2/M inhibitor nocodazole to understand whether TvCMD1 was expressed specifically in the G2/M phase [21]. The signal for TvCMD1 was almost undetected in logarithmic-phase and cold-synchronized cells. Notably, a time-dependence for TvCMD1 was exhibited in nocodazole-treated cells (Fig. 5). This result provided a positive correlation between the G2/M phase and TvCMD1 level in *T. vaginalis*.

**Fig. 6:**
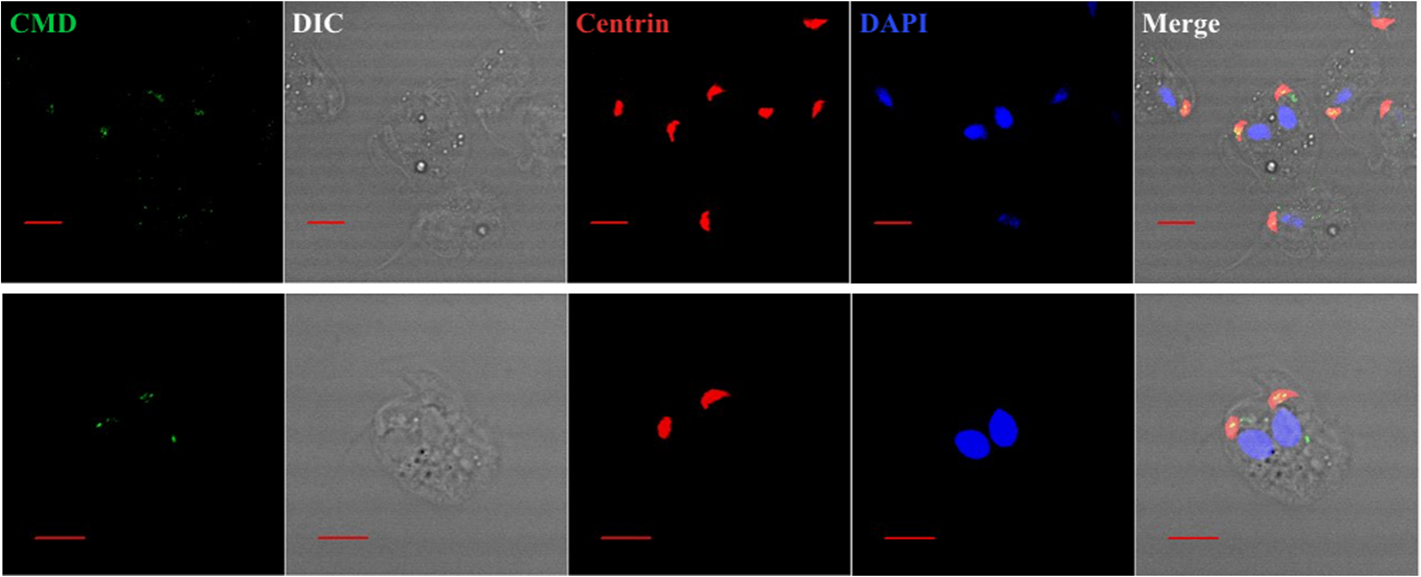
Transportation of TvCMD1 in dividing cells. Immunofluorescence analysis shows the distribution of TvCMD1 in di-nucleated dividing cells. TvCMD1 is shown in green, basal body in red and the nucleus in blue. The images showed that TvCMD1 was expressed and translocated to/ from the basal body when cells underwent cytokinesis.* Scale-bars*: 5 μm

A further assessment of the TvCMD1 role in cell division was monitored by IFA. The trichomonad cells in the logarithmic and stationary phases were collected for observing the abundance and localization of TvCMD1. A weak signal for TvCMD1 was found in the basal body of logarithmic phase cells, as shown in Fig. 3c. TvCMD1 was expressed and translocated to/from the basal body in dividing cells (Fig. 6). Based on these results, we suggested that TvCMD1 is specifically expressed in the G2/M and cytokinesis phases in *T. vaginalis*.

## Discussion

Iron affects several factors in the cell division cycle of eukaryotic cells [15, 16]. *T. vaginalis* displays a growth arrest-like phenotype under iron-restricted conditions [10, 14], accompanied by pseudocyst formation and flagella internalization [40]. Cell cycle regulation and morphological changes suggest that cytoskeletal rearrangement was a part of the responses induced by iron deficiency. However, no report has yet illustrated how iron regulates these cellular behaviors in *T. vaginalis*. TvCMD1 is an unstudied gene in *T. vaginalis* that is considered to have been acquired by LGT [9]. LGT-derived genes usually play housekeeping roles in unicellular parasites. These transferred genes participate in metabolic processes in *T. vaginalis*, such as iron-sulfur cluster assembly [41]. Nevertheless, LGT-derived genes have not been characterized in detail in *T. vaginalis*.

The well-known canonical CMD participates in the β-ketoadipate pathway, which mediates the degradation of aromatic compounds, yielding acetyl-CoA for energy generation in bacteria, archaea and fungi [3, 33]. No genes for the pathway except CMD were identified in the genome of *T. vaginalis*, though this could be due to the incompleteness of the genome [9].

According to the composition of the dualCMD genes, we believe that dualCMD was intragenically duplicated or fused with genes from the CMD superfamily. Two three-dimensional dualCMD structures of *Rhizobium meliloti* (PDB ID: 4G9Q) and *Paraburkholderia xenovorans* (PDB ID: 2Q0T) are proposed in the protein databank (PDB, http://www.rcsb.org/) [42]. These dualCMDs may exert a similar function in aromatic compound catabolism since *R. meliloti* and *P. xenovorans* have been shown to possess the protocatechuate degradation pathway [43, 44]. However, no report describes the function of these dualCMDs. The canonical CMD presents as a homo-hexamer of 13-kDa subunits in *Pseudomonas putida* and *Legionella pneumophila* [3], whereas the dualCMDs of *R. melilot* and *P. xenovorans* were exhibited as a homo-trimer. The conserved ring configuration was formed by both the trimer and hexamer, implying the importance of oligomerization for function. However, no eukaryotic dualCMD had been analyzed.

Intragenic duplication and gene fusion are crucial forces for provoking genetic diversity and novel gene function [45–47]. Organisms that encode (1) dualCMD only, (2) dualCMD and canonical CMD, and (3) dualCMD, canonical CMD and P3,4O are shown in our investigation. The phylogenetic distribution of dualCMDs revealed a low relevance between TvCMD1 and P3,4O-encoding organisms. Additionally, we ruled out the possible involvement of TvCMD1 in aromatic compound degradation in *T. vaginalis* using protein induction and metabolomics approaches. Gene Ontology (GO) predicted that TvCMD1 functions in electron transport, namely, it has peroxiredoxin activity [48]. Furthermore, non-canonical CMD has been demonstrated to have disulfide reductase or thioredoxin activities [31, 32]. The putative cysteine residues were not found in the protein sequence, suggesting that neither electron transfer nor iron-sulfur cluster docking was employed by TvCMD1.

Steady-state expression of TvCMD1 was only detected in mRNA, rather than at the protein level in the logarithmic phase of this protist. This suggests a high turnover rate of the TvCMD1 protein in actively proliferating cells. Expression of TvCMD1 could be induced by stresses that slow the growth rate in *T. vaginalis* [29, 36–39]. Additionally, the basal body assembled the axostyle-pelta complex that involved in the separation of daughter cell in cytokinesis [17, 18], implying that basal body-localized TvCMD1 is related to cell proliferation. *T. vaginalis* has a unique cell cycle that lacks ordinary G1 checkpoints and shows a dominant G2/M proportion [21]. In addition, iron deficiency-induced cell cycle arrest at the G2/M phase has been demonstrated in mammalian cells and *T. vaginalis* [15, 16]. We speculated that TvCMD1 might function in mitotic processes since it accumulated when cells were treated with the microtubule inhibitor nocodazole. Moreover, the accumulation of TvCMD1 under stressful circumstances possibly resulted from the blockage of cytokinesis and thus delayed the turnover of TvCMD1. Accordingly, these phenomena further emphasized that TvCMD1 might take part in the regulation of cell proliferation in *T. vaginalis*.

A recent study on the detergent-resistant cytoskeletal components of a Parabasalian protist *Tetratrichomonas gallinarum* [49] identified a series of cytoskeletal proteins but not dualCMD protein within the cytoskeleton fraction. In the present work, we clearly demonstrated that the *T. vaginalis* dualCMD is a water-soluble protein, and the gene expression is induced by iron-deficient culture condition. It is reasonable to assume that the expression of the dualCMD gene in *T. gallinarum* is also regulated by a similar mechanism. These can explain why dualCMD protein was not identified in the detergent-resistant cytoskeletal components of *T. gallinarum*. Moreover, the genome sequence of *T. gallinarum* is not available at this stage; it is still questionable whether dualCMD gene exists in all Trichomonadidae.

*Trichomonas* and *Giardia* share various aspects, such as they possessed mitochondrion-related organelles hydrogenosome and mitosome, respectively for microaerophilic living style [50–52]. Most importantly, these flagellates are considered to be early branching eukaryotes [53] and have genetic materials that were frequently transferred from the Alphaproteobacteria [54]. The distribution of dualCMDs revealed that Proteobacteria is the largest group of dualCMD carriers (407 out of 985). Furthermore, the coexistence of P3,4O and canonical CMD was found in most dualCMD-possessing Proteobacteria (285 out of 407), so we believe that intragenic duplication or a gene fusion event for dualCMD may have taken place in this phylum. A report indicated that genetic fragments of *T. vaginalis* were frequently donated by Firmicutes bacteria [7]. In addition, the incongruent phylogeny and patchy distribution suggested that the dualCMD of unicellular protists underwent at least two transfer steps, from Proteobacteria to Firmicutes and further to unicellular protists [55]. LGT usually occurred while the donor and recipient were in the same microenvironment or habitat [6]. TvCMDs are closely related to *Veillonella * sp., whereas GiCMD is probably acquired from the bacterial genus *Clostridium* and *Bifidobacterium*, indicating that dualCMD transfer occurred in the vaginal and intestinal tract, respectively [56–58]. Surprisingly, our preliminary result showed that GiCMD (GL50581_3192) is also a basal body-associating protein in *G. intestinalis* (unpublished data), further suggesting the evolutionary accordance of dualCMDs in unicellular protists.

It is important to illustrate the evolutionary routes of dualCMDs in flagellates. We propose three theories for how TvCMD1 gained a distinct function from canonical CMDs. (1) As stated in the previous paragraph, because a dualCMD was identified in bacteria that encodes a canonical CMD, it is likely that the canonical CMD was duplicated/ fused in the bacterial lineage before transferring to the flagellates. (2) The flagellates acquired the canonical CMD and discarded it after a gene duplication/ fusion event. *T. vaginalis* encodes a mass of duplicated genes within the genome, and thus, this could be a reason for the three duplicated/ fused CMDs in the protist. (3) Since dualCMDs existed in parallel with canonical CMDs, the function of dualCMDs was not similar to the well-studied CMDs. However, the time point for gene transfer and duplication/ fusion should be defined.

LGT is still a controversial theory for the evolution of eukaryotes. The convenience of next generation sequencing provided whole genome sequencing data and evidence for LGT events within the new annotated metazoan genomes. For instance, the protein-coding genes of marine fish and tardigrade were proposed that had unexpected LGT origin genes derived from bacteria [59, 60]. However, reports of opposite perspective indicated that these LGT origin genes might result from bacterial contamination while collecting DNA/ RNA samples [61, 62]. In this study, we showed that TvCMD1 is a functional LTG gene since the expression level of this gene can be detected in individual RNA sequencing experiments and even in a dataset of expressed sequence tag (EST) [29, 36, 37, 39]. Moreover, the TvCMD1 gene can be identified in the genome of different *T. vaginalis* reference isolates from ATCC (data not shown). Although, LGT-derived genes were not stably maintained in eukaryotic genomes [63]. The continuous gain and loss of LGT-derived genes in eukaryotic lineages implied that these genes might be critical for the close-related protists but not influence other organisms.

## Conclusion

In summary, this is the first characterization of a dualCMD. We excluded the probable involvement of aromatic compound degradation by TvCMD1 using phylogenetic and metabolomics approaches and defined TvCMD1 as a novel basal body-associated protein. TvCMD1 gathered in the basal body upon cell-cycle blockage due to stress, such as iron deficiency, implying that the function of TvCMD1 is related to the progression of cell division in *T. vaginalis.* Most importantly, this study describes a new class of CMD that evolved distinctly from its bacterial ancestor and provides a probable explanation for the importance of LGT in the coevolution of the flagellate *T. vaginalis*.

## Additional files

Additional file 1: Table S1. The dualCMD protein sequences analyzed in this study. (XLSX 187 kb)

Additional file 2: Table S2. Primer sets used for the quantitative real-time PCR. (PDF 32 kb)

Additional file 3: Figure S1. Sequence alignment of dualCMDs in *T. vaginalis*. a Protein sequence alignment of TvCMD1 (TVAG_256720), 2 (TVAG_107080) and 3 (TVAG_474690). Gray color indicates conserved CMD signatures in sequences. b CMD signature alignments against three bacterial CMDs. TvCMD1 was divided into 2 CMD signatures and aligned with the CMD of *Pseudomonas putida* (Q88N35), *Methanosarcina acetivorans* (Q8TJP3 or MA3736) and *Legionella pneumophila* (Q5ZYG6 or lpg0406). The blue box indicates the conserved thioredoxin-like domain (CXXC). The brown boxes indicate cysteine residues responsible for iron-sulfur cluster binding. Asterisks (*) indicates a fully conserved residue at the position; colon (:) indicates a residue with strong similarity; period (.) indicates a residue with weak similarity. (PDF 345 kb)

Additional file 4: Figure S2. TvCMD1 is the dominant isoform in *T. vaginalis* under iron-deficient condition. The expression levels of TvCMDs in trichomonad cells cultured under iron-rich (IR, black bar) and iron-deficient (ID, gray bar) conditions were determined by using quantitative RT-PCR. (PDF 94 kb)

Additional file 5: Figure S3. Cloning, expression of recombinant TvCMD1 and antibody assessment. a TvCMD1 coding sequence was cloned into a pTrcHis-TOPO vector. b The expression of recombinant TvCMD1 (rTvCMD1) protein in *E. coli* was induced by IPTG treatment. c The rabbit anti-TvCMD1 antibody against the native TvCMD1 protein. (PDF 93 kb)

Additional file 6: Figure S4. Phylogenetic association of the dualCMD proteins. The Bayesian consensus tree of dualCMDs was inferred from 2 Monte Carlo Markov Chains (MCMC) chains based on the Le Gascuel model in PhyloBayes. Posterior probabilities (PP) were indicated as circles on nodes, and the size of circles represented the PP values (from ~0.5 to 1). Red circles indicate that the organisms have canonical CMD within their genomes. Red asterisks show organisms that possess protocatechuate 3,4-dioxygenase (P3,4O), the first enzyme of the pathway, and canonical CMD. Actinobacteria are shown in purple; Bacteroidetes are shown in light green; Firmicutes are shown in brown; Proteobacteria are shown in blue. Fungal and protistic dualCMDs are shown in red; archaeon dualCMDs are shown in green. Group 1: Actinobacteria, Bacteroidetes, Firmicutes, Fusobacteria, Proteobacteria, other bacteria. Group 2: Archaea, Bacteroidetes, Firmicutes, Fusobacteria, Proteobacteria, other bacteria. (PDF 124 kb)

## Abbreviations

Ben: benzoate
BSA: bovin serum albumin
CMD: γ-Carboxymuconolactone decarboxylase
DAPI: 4′,6-diamidino-2-phenylindole
DIP: dipyridyl
EST: expressed sequence tag
GO: gene ontology
IFA: immunofluorescence assay
IPTG: isopropyl β-D-1-thiogalactopyranoside
LGT: lateral gene transfer
MCMC: Monte Carlo Markov Chains
Pca: protocatechuate
PDB: Protein Data Bank
PI: propidium iodide
SDS-PAGE: sodium dodecyl sulfate polyacrylamide gel electrophoresis
TTBS: Tris-buffered saline with Tween 20
UPLC-MS/ MS: ultra-performance liquid chromatography tandem mass-spectrometry
YI-S medium: yeast extract iron-serum medium
