# Supplementary material for: γ-Carboxymuconolactone decarboxylase: a novel cell cycle-related basal body protein in the early branching eukaryote Trichomonas vaginalis
Source: Parasit Vectors. 2017 Sep 26;10:443. doi: 10.1186/s13071-017-2381-4 (PMC5615479; doi:10.1186/s13071-017-2381-4)
Supplement: Supplementary file 2 — Primer sets used for the quantitative real-time PCR. (PDF 32 kb) [file 13071_2017_2381_MOESM2_ESM.pdf]

Additional file 2: Table S2. Primer sets used for real-time PCR

Oligo name		Sequence
TvCMD1 (TVAG_256720)	Forward	GAGTTACAAAGCGACGGA
	Reverse	TCCAAAGACATTTCTAAAATGATT
TvCMD2 (TVAG_107080)	Forward	TTAGGTCATGGAAAATGGGA
	Reverse	CATTCCCTTAGCAAATTTCA
TvCMD3 (TVAG_474690)	Forward	CTATTTTAATAGGATCAAGAT
	Reverse	TTCTTTTCTATTTTCTTTTGT
