# Supplementary material for: γ-Carboxymuconolactone decarboxylase: a novel cell cycle-related basal body protein in the early branching eukaryote Trichomonas vaginalis
Source: Parasit Vectors. 2017 Sep 26;10:443. doi: 10.1186/s13071-017-2381-4 (PMC5615479; doi:10.1186/s13071-017-2381-4)
Supplement: Supplementary file 3 — Sequence alignment of dualCMDs in T. vaginalis. a Protein sequence alignment of TvCMD1 (TVAG_256720), 2 (TVAG_107080) and 3 (TVAG_474690). Gray color indicates conserved CMD signatures in sequences. b CMD signature alignments against three bacterial CMDs. TvCMD1 was divided into 2 CMD signatures and aligned with the CMD of Pseudomonas putida (Q88N35), Methanosarcina acetivorans (Q8TJP3 or MA3736) and Legionella pneumophila (Q5ZYG6 or lpg0406). The blue box indicates the conserved thioredoxin-like domain (CXXC). The brown boxes indicate cysteine residues responsible for iron-sulfur cluster binding. Asterisks (*) indicates a fully conserved residue at the position; colon (:) indicates a residue with strong similarity; period (.) indicates a residue with weak similarity. (PDF 345 kb) [file 13071_2017_2381_MOESM3_ESM.pdf]

Additional file 3: Figure S1. Sequence alignment of dualCMDs in *T. vaginalis*.

a

```
TvCMD1    MS-YKATEHDIEQNDPEYTAFFKRFELMVKNESKIDDKSRYLGILGALIGAKAIDYFPIILEMSLDNILTPNEVKEVLYEAAYILGIANVFPFLKLTNDIFKAKNINLPLENQSKVAPGE
TvCMD2    MSNYKEVLDELEHNDPEYFEFFYRFEKLVQSETKIDEKNRYLGILGSLIGSKSIDYYPYIIEQVLTKILTPQEIKEVLYEAAFILGASTVYPFLKVTNDIFKQHNINLPLENQCKVSPEE
TvCMD3    MS-NSLPSIDPRIPDAEFLNVFNEFENDVKNEIKVENEYRIPAILAILIGSRSIDYFPTFLDEAVSSGITITQIKEIIYQGIMLIGMAHVYPFLKIVNEHF---NISEPADHHSTVTKEN
          **  .    : .  *.*:  .* .**  *:.* *:::: *  .**. ***:::***:* :::  :  . :*  ::**::*:.  ::* : *:****:.*: *   **. * ::::..*:  :

TvCMD1    GLQKGIEKQAEAFGPGMEKFHTMGPMAKWVVSFMYGEFYTREVLTVKERELVLSCIFIGHGDAAPQFNQHIKANIHVGNNKKFLIQMILSLVPYLGYPRTFNALKVLND---AIPDE---
TvCMD2    ALKKGIEKQKESFGPGMEDFHTKGPMNKWVVTFMYGQLYTREVLTVKQREIVITCIFIGYGDATPQFLQHVKALTNLGHGKWDLIQMILSLVPYIGYPRTFNALRAINE---AVPDPLYM
TvCMD3    RKEKGIEKLNESFGFIMNGLPGLGPMSDWLMKYYYGELYTRDILSMRQRELILLCFLSVHGDSYMALSAHVMSNINVGNDKEFLKNVFYSLVPYMGSVKAYNAIKCIDDWKSPVHSD---
            :*****  *:**  *: :   *** .*::.: **::***::*::::**::: *::  :**:   :  *: :  ::*:.*  * ::: *****:*  :::**:: :::   .: .

TvCMD1    ---------------
TvCMD2    KVMKFAKGMYGSIFG
TvCMD3    ---------------
```

b

```
TvCMD_1-110                 MSYKATE---------------------HDIEQN---DPEYTAFFKR-FE
Q88N35_P.putida             MDEKQRYDAGMQVRRAVLGDAHVDRSL-EKLNDF---NGEFQEMITRHAW
Q8TJP3_M.acetivorans        MTEHEEILESMGEKM-----GFTPQVL-ETLGDL---DPEFLHKYRR-CD
Q5ZYG6_L.pneumophila        MSDKFSH---------------ITKDITTQLAKFRKEMPELMTGFSS-LA
                            *  :                          : .       *

TvCMD_1-110                 LMVKNESKIDDKSRYLGILGALIGAKAIDYFPIILEMSLDNILTPNEVKE
Q88N35_P.putida             GDIWTRPGLPRHTRSLITIAMLIGMNRNDELKLHLRAAANNGVTRDEIKE
Q8TJP3_M.acetivorans        HKILTDGALPSKVKILMALAVVASKQCESCTVAQMKSALKNGATKEEIME
Q5ZYG6_L.pneumophila        QAATKDGALDKKTKELIAMALAVAKQCPGCIGFHSQTLVKLQATREELLE
                                .   :  : : *  :.   . :  .      .   .   * :*: *

TvCMD_1-110                 VLYEAAYILGIANVFPFLKLTNDIFKAKNINLPLE
Q88N35_P.putida             VLMQSAIYCGIPAANATFHLAESVWDELGVESR-Q
Q8TJP3_M.acetivorans        TMEVIFITSGAPAVAACRNALKM-LKD--M-----
Q5ZYG6_L.pneumophila        TLGMAVYMGGGPSLMYAAEALEA-FEEFSK-----
                            .:        * .       .   :    .

TvCMD1_111-233              NQSKVAPGEGLQKGIEKQAEAFG-PGM-EKFHTM-GPMAKWVVSF--MYG
Q88N35_P.putida             MDEKQRYDAGMQVRRAVLGDAHVDRSL-EKLNDFNGEFQEMITRH--AWG
Q8TJP3_M.acetivorans        MTEHEEILESMGEK-----MGFTPQVL-ETLGDLDPEFL---HKYRRCDH
Q5ZYG6_L.pneumophila        MSDKFSH---------------ITKDITTQLAKFRKEMPELMTGFSSLAQ
                             .:                         :    :   :   :       .

TvCMD1_111-233              EFYTREVLTVKERELVLSCIFIGHGDAAPQFNQHIKANIHVGNNKKFLIQ
Q88N35_P.putida             DIWTRPGLPRHTRSLITIAMLIGMN-RNDELKLHLRAAANNGVTRDEIKE
Q8TJP3_M.acetivorans        KILTDGALPSKVKILMALAVVASKQ-CESCTVAQMKSALKNGATKEEIME
Q5ZYG6_L.pneumophila        AATKDGALDKKTKELIAMALAVAKQ-CPGCIGFHSQTLVKLQATREELLE
                              .    *  : : *:  .:  .            : ::  :   .:. : :

TvCMD1_111-233              MILSLVPYLGYPRTFNALKVLNDAIPD-------E
Q88N35_P.putida             VLMQSAIYCGIPAA-NATFHLAESVWDELGVESRQ
Q8TJP3_M.acetivorans        TMEVIFITSGAPAV-AACRNALKMLKDM-------
Q5ZYG6_L.pneumophila        TLGMAVYMGGGPSL-MYAAEALEAFEEF-SK----
                             :        * *         . . :
```

a Protein sequence alignment of TvCMD1 (TVAG_256720), 2 (TVAG_107080) and 3 (TVAG_474690). Gray color indicates conserved CMD signatures in sequences. **b** CMD signature alignments against three bacterial CMDs. TvCMD1 was divided into 2 CMD signatures and aligned with the CMD of *Pseudomonas putida* (Q88N35), *Methanosarcina acetivorans* (Q8TJP3 or MA3736) and *Legionella pneumophila* (Q5ZYG6 or lpg0406). The blue box indicates the conserved thioredoxin-like domain (CXXC). The brown boxes indicate cysteine residues responsible for iron-sulfur cluster binding. Asterisks (*) indicates a fully conserved residue at the position; colon (:) indicates a residue with strong similarity; period (.) indicates a residue with weak similarity.
