# Supplementary material for: γ-Carboxymuconolactone decarboxylase: a novel cell cycle-related basal body protein in the early branching eukaryote Trichomonas vaginalis
Source: Parasit Vectors. 2017 Sep 26;10:443. doi: 10.1186/s13071-017-2381-4 (PMC5615479; doi:10.1186/s13071-017-2381-4)
Supplement: Supplementary file 4 — TvCMD1 is the dominant isoform in T. vaginalis under iron-deficient condition. The expression levels of TvCMDs in trichomonad cells cultured under iron-rich (IR, black bar) and iron-deficient (ID, gray bar) conditions were determined by using quantitative RT-PCR. (PDF 94 kb) [file 13071_2017_2381_MOESM4_ESM.pdf]

Additional file 4: Figure S2. TvCMD1 is the dominant isoform in *T. vaginalis* under iron-deficient condition.

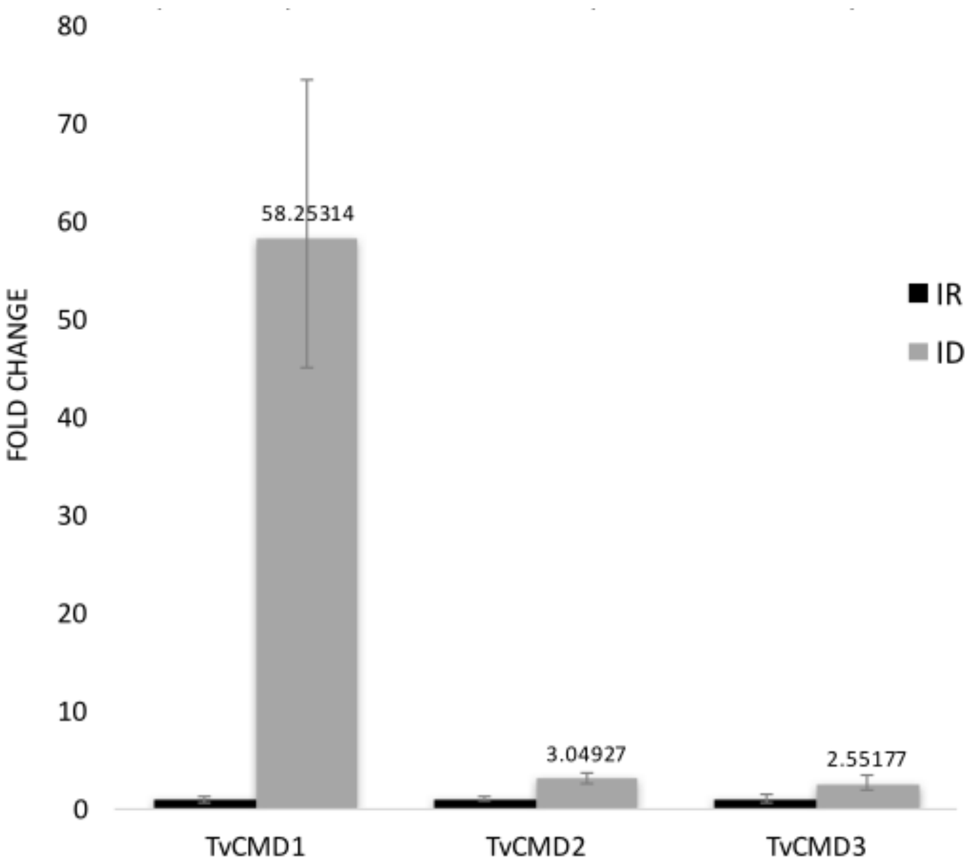

The expression levels of TvCMDs in trichomonad cells cultured under iron-rich (IR, black bar) and iron-deficient (ID, gray bar) conditions were determined by using quantitative RT-PCR.
