# Supplementary material for: γ-Carboxymuconolactone decarboxylase: a novel cell cycle-related basal body protein in the early branching eukaryote Trichomonas vaginalis
Source: Parasit Vectors. 2017 Sep 26;10:443. doi: 10.1186/s13071-017-2381-4 (PMC5615479; doi:10.1186/s13071-017-2381-4)
Supplement: Supplementary file 5 — Cloning, expression of recombinant TvCMD1 and antibody assessment. a TvCMD1 coding sequence was cloned into a pTrcHis-TOPO vector. b The expression of recombinant TvCMD1 (rTvCMD1) protein in E. coli was induced by IPTG treatment. c The rabbit anti-TvCMD1 antibody against the native TvCMD1 protein. (PDF 93 kb) [file 13071_2017_2381_MOESM5_ESM.pdf]

Additional file 5: Figure S3. Cloning, expression of recombinant TvCMD1 and antibody assessment.

a

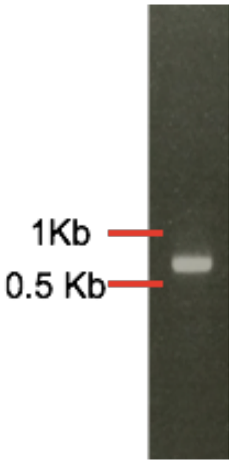

b

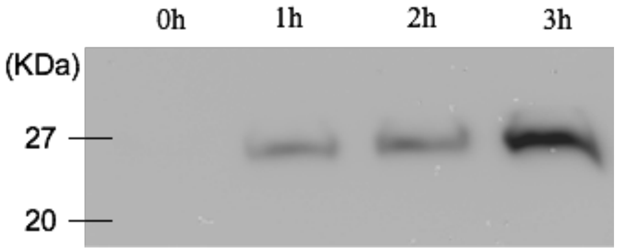

c

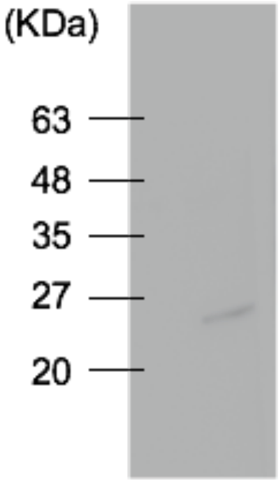

a TvCMD1 coding sequence was cloned into a pTrcHis-TOPO vector. **b** The expression of recombinant TvCMD1 (rTvCMD1) protein in *E. coli* was induced by IPTG treatment. **c** The rabbit anti-TvCMD1 antibody against the native TvCMD1 protein.
