# Supplementary material for: γ-Carboxymuconolactone decarboxylase: a novel cell cycle-related basal body protein in the early branching eukaryote Trichomonas vaginalis
Source: Parasit Vectors. 2017 Sep 26;10:443. doi: 10.1186/s13071-017-2381-4 (PMC5615479; doi:10.1186/s13071-017-2381-4)
Supplement: Supplementary file 6 — Phylogenetic association of the dualCMD proteins. The Bayesian consensus tree of dualCMDs was inferred from 2 Monte Carlo Markov Chains (MCMC) chains based on the Le Gascuel model in PhyloBayes. Posterior probabilities (PP) were indicated as circles on nodes, and the size of circles represented the PP values (from ~0.5 to 1). Red circles indicate that the organisms have canonical CMD within their genomes. Red asterisks show organisms that possess protocatechuate 3,4-dioxygenase (P3,4O), the first enzyme of the pathway, and canonical CMD. Actinobacteria are shown in purple; Bacteroidetes are shown in light green; Firmicutes are shown in brown; Proteobacteria are shown in blue. Fungal and protistic dualCMDs are shown in red; archaeon dualCMDs are shown in green. Group 1: Actinobacteria, Bacteroidetes, Firmicutes, Fusobacteria, Proteobacteria, other bacteria. Group 2: Archaea, Bacteroidetes, Firmicutes, Fusobacteria, Proteobacteria, other bacteria. (PDF 124 kb) [file 13071_2017_2381_MOESM6_ESM.pdf]

Additional file 6: Figure S4. Phylogenetic association of the dualCMD proteins.

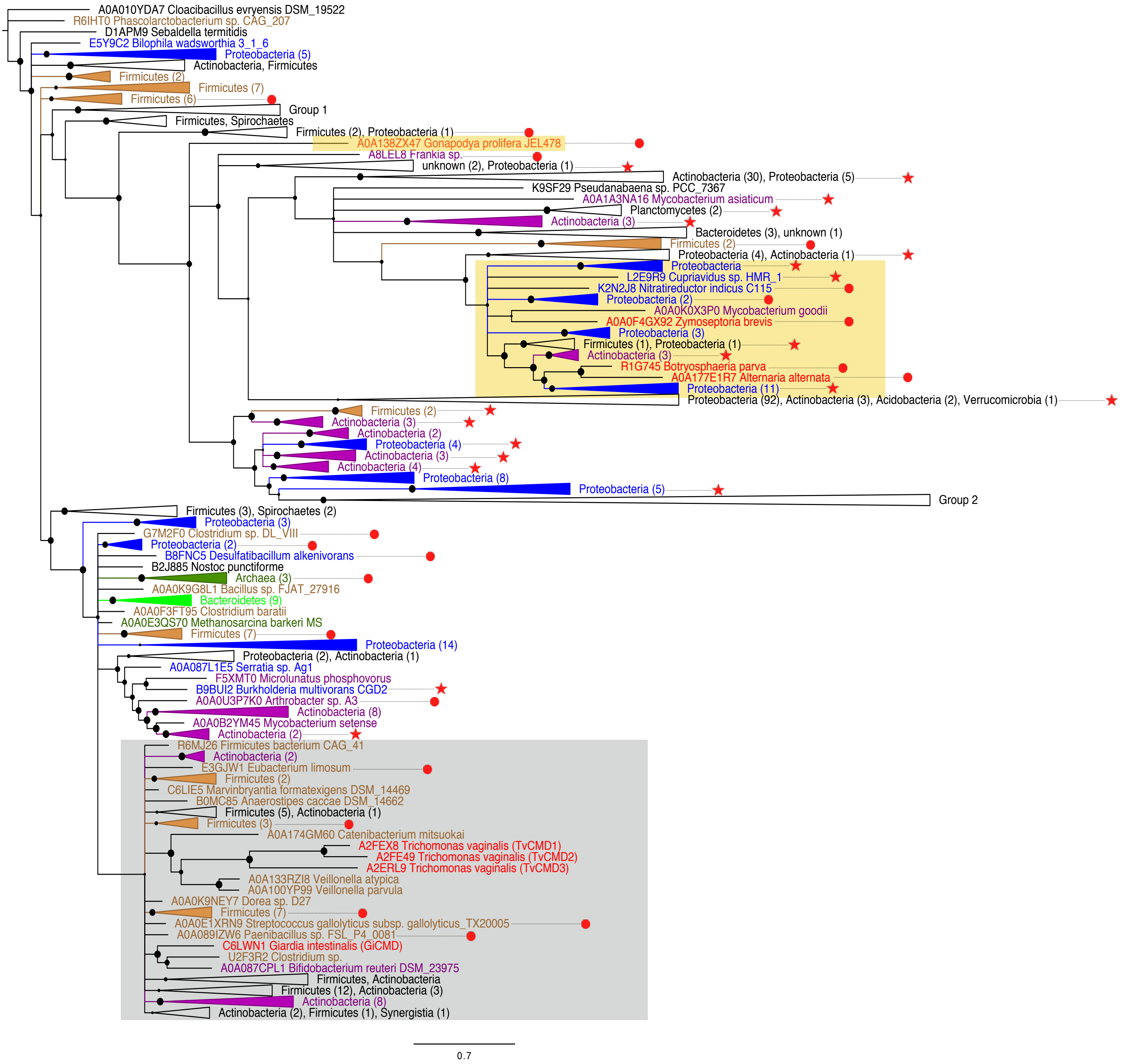

The Bayesian consensus tree of dualCMDs was inferred from 2 Monte Carlo Markov Chains (MCMC) chains based on the Le Gascuel model in PhyloBayes. Posterior probabilities (PP) were indicated as circles on nodes, and the size of circles represented the PP values (from ~0.5 to 1). Red circles indicate that the organisms have canonical CMD within their genomes. Red asterisks show organisms that possess protocatechuate 3,4-dioxygenase (P3,4O), the first enzyme of the pathway, and canonical CMD. Actinobacteria are shown in purple; Bacteroidetes are shown in light green; Firmicutes are shown in brown; Proteobacteria are shown in blue. Fungal and protistic dualCMDs are shown in red; archaeon dualCMDs are shown in green. Group 1: Actinobacteria (11), Bacteroidetes (6), Firmicutes (4), Fusobacteria (2), Proteobacteria (4), other bacteria (2). Group 2: Archaea (5), Bacteroidetes (31), Firmicutes (4), Fusobacteria (2), Proteobacteria (88), other bacteria (3).
